# Effect of soy isoflavones supplementation on migraine characteristics, mental status and calcitonin gene-related peptide (CGRP) levels in women with migraine: results of randomised controlled trial

**DOI:** 10.1186/s12937-022-00802-z

**Published:** 2022-07-30

**Authors:** Maedeh Babapour, Fariborz Khorvash, Mohammad Hossein Rouhani, Abed Ghavami, Hatav Ghasemi‐Tehrani, Zahra Heidari, Mojtaba Karbasi, Fatemeh Moradi, Gholamreza Askari

**Affiliations:** 1 Department of Community Nutrition, School of Nutrition and Food Science, Isfahan University of Medical Sciences, Isfahan, Iran; 2 Neurology Research Center, School of Medicine, Isfahan University of Medical Sciences, Isfahan, Iran; 3 Department of Reproductive, Shahid Beheshti Fertility and Infertility Clinic, Isfahan University of Medical Sciences, Isfahan, Iran; 4 Department of Biostatistics and Epidemiology, School of Health, Isfahan University of Medical Sciences, Isfahan, Iran; 5 Department of English Language, Faculty of Information and Medical Informatics, Isfahan University of Medical Sciences, Isfahan, Iran

**Keywords:** Migraine, Headache, Soy Isoflavones, CGRP

## Abstract

**Background:**

Literature suggests a relationship between estrogen levels and migraine headache pathogenesis. However, the effect of soy isoflavones on migraine characteristic remains unclear. This study aimed to investigate the effect of soy isoflavones on migraine characteristics and calcitonin gene-related peptide (CGRP) levels in women with migraine.

**Methods:**

Eighty-three participants completed a randomized double-blind controlled trial, receiving 50 mg per day soy isoflavones or placebo supplementation for 8 weeks. Migraine severity, migraine days per month, frequency and duration of attacks, mental status, quality of life and serum CGRP levels were measured at baseline and the end of the intervention. Bivariate comparison and intention-to-treat (ITT) were used for analysis.

**Results:**

Soy isoflavones intake resulted in a significant decrease in mean frequency (-2.36 vs -0.43, *P* < 0.001), duration (-2.50 vs -0.02, *P* < 0.001) of migraine attacks and CGRP level (-12.18 ng/l vs -8.62, *P* = 0.002) in compared to placebo group. Also, a significant improvement was found in quality of life (16.76 vs 2.52, *P* < 0.001). Although, reduction in the migraine severity and mental status did not reach a statistically significant level (*P* > 0.05).

**Conclusion:**

soy isoflavones supplementation may be considered as a complementary treatment for women with migraine to improve migraine characteristics and reduce the burden of disease.

## Introduction

Migraine is considered as an intense unilateral throbbing headache which often accompanied by nausea, vomiting, photophobia and phonophobia, which can be exacerbated by routine physical activity [1]. Untreated migraine may last for about 4 to 72 h [2]. Migraine is a highly burdensome disease that affects more than 15 percent of the world’s global population [3, 4] and Approximately, 14% in Iranian population Also, affecting individuals who are predominantly female about three times higher in females [5].

Migraine management is often challenging. Most commonly prescribed migraine prophylactic drugs such as beta-blockers, calcium-channel blockers, anticonvulsants, and antidepressants that were initially designed to treat other conditions, and subsequently found to be effective in migraine [6]. Although the efficacy and adverse effects are rather unfavorable. Many patients would be willing to take them if they were fully informed about their side effects [7]. Literature shows a more rate of depression and anxiety in these patients that induce a poor quality of life and disability in migraine patients [8].

Migraine physiopathology is still chiefly unknown. But in both vascular and neuronal theories regarding the cause and nature of migraine attacks, a possible role for inflammation is considered [9]. It has been shown that cytokines levels are altered in migraineurs, inflammatory mediators can stimulate calcitonin gene-related peptide (CGRP) transcription [10]. CGRP is a 37 amino acid neuropeptide, predominantly produced in up to 50% of trigeminal neurons of the nervous system and released by perivascular nerve endings following trigeminal nerve activation. CGRP acts as a potent dilator of peripheral and cerebral blood vessels [11]. Evidences suggest that CGRP concentration correlates with migraine and pain; thus, an integral role has been considered for CGRP in the pathophysiology of migraine. According to outstanding property sets of CGRP and this point that widely accepted migraine headache is generated by activation of the trigeminovascular system, the major pain-signaling system of the viscera brain CGRP receptor antagonists is a new target in migraine treatment [12].

The use of nutraceuticals, vitamins and mineral butterbur is expanding in migraineurs due to their potential effect for migraine relief, and minimal side effects and drug dependence [13]. Isoflavones belong to a class of diphenol compounds known as phytoestrogens and have structural and functional similarities to the human estrogen. Considerable evidence suggests a link between estrogen and migraine. Most dietary source contains a mixture of derivative based on three isoflavones genistein, daidzein and glycitein [14]. Epidemiological studies over the last decades have suggested protective effects of isoflavones family against a number of chronic disease, including coronary heart disease and breast, endometrial, and prostate cancers [15]. Literature show the number of migraine attacks is significantly higher during the end of the luteal phase when estrogen levels are low Also, severity and frequency of migraine attacks decreases in women with menstrual migraines. It improves the quality of life following isoflavones consumption too [16].

According to the beneficial impacts of isoflavones on health as well as lack of enough study to evaluate the effects of soy isoflavones on migraine headache patients, the present study aimed to investigate the effects of soy isoflavones supplementation on migraine headache characteristics, mental status, quality of life and CGRP concentration in adult women with migraine.

## Method & martials

### Trial design

This study was a double-blind, placebo-controlled clinical trial, registered at the Iranian Registry of Clinical Trials website (IRCT, www.irct.ir) with the Ref. No IRCT20121216011763N44 and the CONOSRT guidelines were used for clinical trial reporting [17]. An information sheet was provided to participants and signed informed consent was obtained prior to the study. Approval was obtained from the Ethics Committee of the Isfahan University of Medical Sciences (approval number: IR.MUI.RESEARCH.REC.1398.532).

### Inclusion & exclusion criteria

Women were enrolled from two neurology clinics (Imam Moosa Sadr and Khurshid) affiliated with the Isfahan University of Medical Sciences, Isfahan, Iran, between January 2020 and March 2020. Patients were eligible to participate if they were more than 18 years old until menopause/perimenopause ages diagnosed as having migraine by a neurologist (F.K) based on ICDH-3 criteria [18]; and willing to participate in the trial.

Exclusion criteria were: women with menopause/perimenopause symptoms or/and endometrial and breast cancer, ovarian cysts, uterine fibroids and uterine polyps diagnosed by a gynecologist (H.G); pregnancy or intention to pregnancy and lactation; having other neurologic or endocrine comorbidities (thyroid disease, cardiovascular disease, high blood pressure, diabetes, cancer, hepatic or renal disorders); taking any nutritional or herbal supplements; having a history of anti-inflammatory or contraceptives drugs at least three months prior to the study; diagnosed as having migraine with aura or other types of headache such as headache due to menstrual cycle tension-type headaches, trigeminal autonomic cephalagia, tension-type headache, medication overuse headache, and cluster headache. Women were also excluded if they adopted a specific physical activity and/or a dietary regimen, severe side effects from the treatment, or poor compliance (less than 80%).

### Sample size

Sample size was determined based on the parallel-two group randomized clinical trial formula with study power of 80%, confidence interval of 95% and based on the severity of attacks [19]. A total of 80 participants (n = 40 per group) was required which was increased to 44 to accommodate a probable 10% dropout rate.

### Randomization and blinding

A stratified (based on age and experimental group) permuted block randomization (with block size 4) was performed using a random number table to randomly allocating participants into intervention or placebo groups (1:1). Placebo and isoflavones supplements were boxed and labelled by the supplement manufacturer to ensure blinding. Participants, investigators and laboratory staff were blinded to the treatment assignment.

### Intervention

Patients in the intervention group received one tablet containing 50 mg isoflavones (Standardized based on 20–27 mg genistein). Placebo group received starch tablets with similar weight and appearance to intervention tablets. Participants in both groups were instructed to consume one tablet a day for eight weeks. Based on previous studies, a dose of 50 mg was sufficient to induce beneficial effects, with no significant gastrointestinal discomfort or adverse side effects [20]. Participants were provided with a sufficient supply of supplements at baseline and each follow-up visit. Isoflavones and placebo supplements were produced and packaged by Goldaru pharmaceutical company, Isfahan, Iran. The investigators and participants remained blinded to treatment allocation. To increase compliance rate, all patients received weekly phone calls during the two months of the intervention. They returned unused supplement packages as an estimate of compliance. The compliance was calculated based on the following formula: Compliance rate: (Tablet taken/Tablet prescribed) *100. All participants were instructed to continue their routine physical activity, dietary intake, and medication throughout the study but not to consume any soy products. They were also advised to inform the researchers of any changes to their therapy or adverse effects of the supplements.

### Assessment of migraine attacks characteristic

Migraine attack characteristics of participants at baseline and the end of the intervention were collected using a self-administered 30-day headache diary. Verbal and written instructions on how to complete the diary were provided. Participants were asked to record the time of migraine attack onset, duration, and severity of the attack (scored from 0 “no pain” to 10 “the worst imaginable pain” based on a visual analog scale (VAS) after each migraine attack regardless of the time of day. The frequency of attack was also recorded as the number of headaches per month. Clinical symptoms of migraine including migraine index (MI), headache diary result (HDR) and migraine headache index score (MHIS) were then calculated using the following formula: MI = frequency × severity; HDR = frequency × duration; and MHIS = frequency × duration × severity [21].

### Assessment of mental status & quality of life assessment

To assess the quality of life of migraine patients, the study completed the migraine-specific quality of life (MSQ) questionnaire for each patient. It included 14 questions and assessed the quality of life of patients during the previous month. Each question was scored with a six-choice answer categorized as 1 (never) to 6 (always). All responses were summed to compute an overall score ranging from 14 to 84. To facilitate interpretation, scores were transformed to a scale of 0 to 100 and then they were reduced from 100. Finally, higher scores indicated a better quality of life The validity of the questionnaire has been examined by Zandifar et al. in Iran [22].

DASS (Depression Anxiety and Stress Scale) questionnaire was completed for each person to assess depression, stress, and anxiety. This questionnaire had 21 phrases. Each subscale of depression, stress and anxiety was evaluated by 7 different phrases. The questionnaire had been validated in previous studies[23]

### Blood collection and biochemical measurements

A 10 ml blood sample was collected from each participant by a trained phlebotomist at the baseline and end of the intervention. After centrifuging the blood samples for 10 min at 2500 rpm (Beckman Avanti J-25; Beckman 174 Coulter, Brea, CA, USA) at room temperature, serum samples were stored at -80ºC until analysis. The serum levels of CGRP was measured by ELISA (Crystal Day Bio-Tec, China).

### Body weight and blood pressure assessment

After screening based on eligibility criteria, participants were asked to complete a questionnaire on the sociodemographic and medical history. Body weight and height were determined with a digital Seca scale (Saca 831, Hamburg, Germany) and a portable stadiometer (Seca, Hamburg, Germany), respectively. Body mass index (BMI) was calculated as weight (kg) divided by the square of the height (m^2^). SBP and DBP measurements were made, in the right arm, with a conventional mercury sphygmomanometer and an appropriately sized cuff, after the participant seated and rested for 10 min. Two measurements were done at least 30 s apart and the average of the two measurements was used for analyses.

### Assessment of diet and physical activity levels

A reliable and valid 3-day food record including two weekdays and 1 weekend during a week, was collected from each participant at baseline and the end of the study to calculate the total energy and nutrients intake relative to migraine. A trained nutritionist blinded to the treatment assignment instructed participants on how to complete their 3-day food record. Dietary intake was then converted to gram/day using Iranian Household Measures. To calculate daily energy and nutrient intake, the Nutritionist IV software (based on the US National Nutrient Databank) modified for Iranian foods was used. The validated Iranian version of the international physical activity questionnaire (IPAQ), was used to compute the level of PA [24]. PA levels were reported as metabolic equivalent hours per day (MET/h/day).

### Statistical analysis

The analyses were conducted on the basis of an intention-to-treat (ITT) approach by using the last value carried forward (LOCF) protocol. The ITT analysis included all patients randomly assigned to isoflavones and placebo groups, regardless of their level of adherence to the trial or withdrawal. Following LOCF protocol, the last available measurements for individuals prior to withdrawal from the study were included in the analysis [25]. Shapiro Wilk test was used to assess the normal distribution of data. Data was reported as mean and Standard Deviation (SD) (for continuous data) and frequency and percentage (for categorical data). To compare qualitative variables, the chi-square test was used and expressed as a number (%). To identify between-group differences, independent sample t tests and for within-group differences paired-sample t tests were performed. All quantitative variables were shown as mean ± SD. Analysis of covariance (ANCOVA) was performed to detect differences between two groups adjusted for baseline value. A *P*-value < 0.05 was considered statistically significant. All statistical analyses of the trial were done by Statistics specialist (Z.H) using SPSS software version 22 (SPSS Inc., Chicago, Illinois).

## Result

**Fig. 1:**
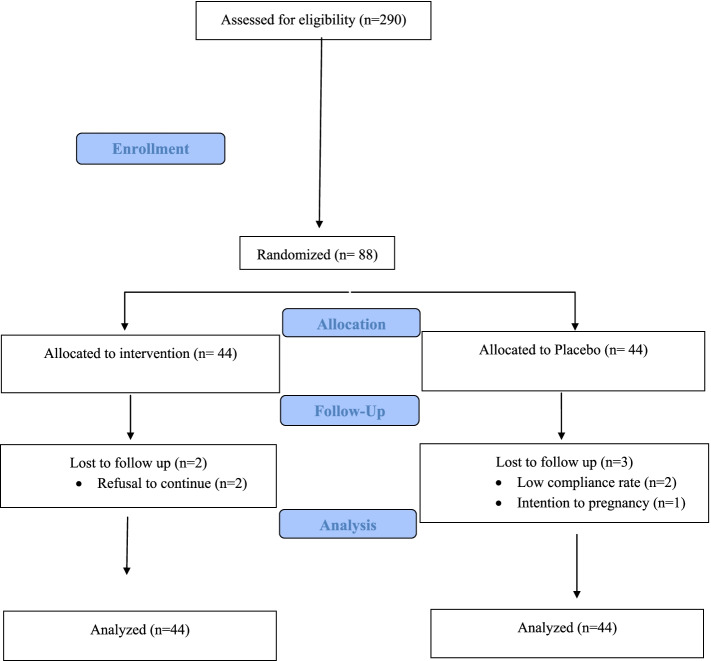
Flow diagram of study

As shown in the study flow diagram (Fig. 1), of 200 women screened for eligibility, eighty- eight women who met the inclusion criteria were included, of whom eighty -five participants completed the 8-week trial. Overall, two women in treatment group and three in the placebo group withdrew before completing the intervention. The reasons for not completing the trial were refusal to continue (*n* = 2) in isoflavones group and low compliance rate (*n* = 2) and intention to pregnancy (*n* = 1) in placebo group. All 88 participants were included in the final statistical analyses using the ITT approach. No adverse effects were reported following isoflavones supplementation.

At the beginning of the study, baseline characteristics of migraine patients including age, anthropometric indices such as height, weight and BMI, blood pressure parameters (systolic blood pressure (SBP) and diastolic blood pressure (DBP), disease duration and sleep time, marital status, education, migraine family history, migraine type, and current medication were not significantly different between the two groups Table 1.

**Table 1:** Baseline characteristic of study subjects

P ^a^	Placebo group (*n* = 44)	Soy isoflavones group (*n* = 44)	Variable		
0.212	35.72 ± 5.61	33.77 ± 8.63	Age (years)		
0.181	73.28 ± 12.15	69.74 ± 12.50	Weight (kg)		
0.736	1.61 ± 0.06	1.60 ± 0.05	Height (m)		
0.244	28.13 ± 4.33	26.97 ± 4.91	BMI (Kg/m2)		
0.671	118.63 ± 2.20	118.38 ± 3.21	SBP (mmHg)		
0.079	77.15 ± 4.93	79.09 ± 5.25	DBP (mmHg)		
0.073	3.46 ± 2.01	4.28 ± 2.21	Disease duration (year)		
0.760	7.38 ± 2.04	7.25 ± 2.12	Sleep duration (hour)		
0.081	6 (13.6)	13 (29.5)	Single		Marital status n (%)
	34 (77.3)	31 (70.5)	Married		
	3 (6.8)	0 (0)	Divorced		
	1 (2.3)	0 (0)	Widow		
0.198	28 (63.4)	21 (47.7)	Yes		Family history n (%)
	16 (36.4)	23 (52.3)	No		
0.739	38 (86.4)	40 (90.9)	Episodic		Migraine type n (%)
	6 (13.6)	4 (9.1)	Chronic		
0.519	27 (61.4)	23 (52.3)	University graduated		Education status n (%)
	17 (38.6)	21 (47.7)	Diploma & under diploma		
0.388	23 (52.3)	28 (63.6)	Yes	NSAIDs	Medication n (%)
	21 (47.5)	16 (36.4)	No		
1	6 (13.6)	7 (15.9)	Yes	Sodium valproate	
	38 (86.4)	37 (84.1)	No		
0.713	3 (6.8)	5 (11.4)	Yes	Benzodiazepine	
	41 (93.2)	39 (88.6)	No		
0.783	7 (15.9)	9 (20.5)	Yes	Beta blockers	
	37 (84.1)	35 (79.5)	No		
0.637	11 (25)	14 (31.8)	Yes	Gabapentin	
	33 (75)	30 (68.2)	No		
1	4 (9.1)	4 (9.1)	Yes	Triptans	
	40 (90.9)	40 (90.9)	No		
0.266	6 (13.6)	2 (4.5)	Yes	SSRIs	
	38 (86.4)	42 (95.5)	No		
1	3 (6.8)	3 (6.8)	Yes	TCAs	
	3 (6.8)	3 (6.8)	No		

Quantitative variables are expressed as mean ± SD and qualitative variables expressed as n (%)

^a^ *p* values resulted from independent t tests for quantitative and Chi-square for qualitative variables between the two groups

Abbreviation: *BMI* Body mass index, *DBP* Diastolic blood pressure, *NSAIDs* Nonsteroidal anti-inflammatory drugs, *SBP* Systolic blood pressure, *SSRIs* Selective serotonin reuptake inhibitor, *TCA* Tricyclic Antidepressants

Base on the 3-d dietary records, no significant differences were observed in dietary intakes of macro-nutrients and micro-nutrients related to migraine also, physical activity (*P* > 0.05) among two groups as presented in Table 2.

**Table 2:** Dietary intake and physical activity of the participants throughout the study

Variable	Soy isoflavones group (*n* = 44)	Placebo group (*n *= 44)	P ^a^
Energy (kcal/day)	2215.9 ± 410/77	2367.13 ± 394.35	0.080
Carbohydrate (g/day)	347.75 ± 79.00	358.95 ± 75.76	0.372
Protein (g/day)	78.07 ± 20.99	81.36 ± 17.18	0.499
Fat (g/day)	67.67 ± 15.22	73.06 ± 13.85	0.098
Vitamin A (μg/day**)**	473.89 ± 313.73	499.65 ± 267.12	0.678
Vitamin D (μg/day)	1.16 ± 0.88	0.99 ± 0.46	0.251
Vitamin E (mg/day)	14.08 ± 5.87	13.73 ± 5.25	0.802
Vitamin C (mg/day)	106.23 ± 79.28	79.12 ± 11.04	0.458
Thiamin (mg/day)	1.89 ± 0.52	1.97 ± 0.46	0.409
Riboflavin (mg/day)	1.70 ± 0.51	1.90 ± 0.51	0.078
Niacin (mg/d)	23.58 ± 3.81	24.64 ± 4.9	0.213
Cobalamin (μg/day**)**	34.53 ± 11.48	37.13 ± 13.37	0.330
Magnesium (mg/day)	232.25 ± 104.55	215.48 ± 77.95	0.982
Physical activity (MET/h/day)	24.13 ± 5.2	23.11 ± 6.36	0.412

Variables are expressed as mean ± SD

^a^ Obtained from independent t test

*MET* Metabolic equivalent task

At the end of the trial, the soy isoflavones group had a significant reduction in migraine attacks characteristic including frequency (mean change: -2.36 ± 1.20 versus -0.43 ± 1.35, *P* < 0.001) and duration (mean change: -2.50 ± 1.74 versus -0.02 ± 2.15, *P* < 0.001). Although severity of migraine headache had not a significant reduction in compared to placebo group (mean change: -0.93 ± 1.95 versus -0.31 ± 1.61, *P* = 0.189). And also, all of migraine indices including MI, HDR and MHIS) had a significant reduction following soy isoflavones supplementation (*P* < 0.05) as reported in Table 3.

**Table 3:** The effects of 8 weeks’ soy isoflavones supplementation on migraine characteristics and clinical indices of migraine

**Soy isoflavones group (n = 44)**					Placebo group (*n* = 44)				P ^b^	P ^c^
	Baseline	End of trial	Change	P ^a^	Baseline	End of trial	Change	P ^a^		
**Characteristics of migraine attack**										
Attacks frequency ( per month)	7.65 ± 2.44	5.29 ± 1.95	-2.36 ± 1.20	< 0.001	7.06 ± 1.43	6.63 ± 1.90	-0.43 ± 1.35	0.040	0.002	< 0.001
Severity	7.06 ± 1.63	6.13 ± 1.78	-0.93 ± 1.95	0.003	6.65 ± 1.62	6.34 ± 1.66	-0.31 ± 1.61	0.197	0.580	0.189
Attacks duration (hr) ^1^	10.38. ± 4.35	7.88 ± 3.50	-2.50 ± 1.74	< 0.001	10.61 ± 2.08	10.63 ± 2.80	-0.02 ± 2.15	0.944	< 0.001	< 0.001
**Clinical indices of migraine**										
MI (frequency * severity)	55.77 ± 26.10	32.02 ± 14.65	-23.75 ± 18.19	< 0.001	47.29 ± 15.94	42.75 ± 17.87	-4.54 ± 12.89	0.024	0.003	< 0.001
HDR (frequency * duration)	80.04 ± 41.23	42.84 ± 26.29	-37.20 ± 22.38	< 0.001	74.79 ± 24.23	70.13 ± 24.11	-4.65 ± 20.22	0.134	< 0.001	< 0.001
MHIS (frequency * duration * severity)	19.18 ± 11.24	8.72 ± 5.91	-10.46 ± 7.43	< 0.001	16.59 ± 6.99	15.12 ± 6.90	-1.47 ± 5.25	0.069	< 0.001	< 0.001

Variables expressed as mean ± SD

a Paired t-test was used to compare pre-post tests

b Independent sample t-test was used to compare groups two by two

c Obtained from analysis of covariance in the adjusted models (adjusted for baseline value and certain dietary factors such as vitamin D, thiamin, riboflavin, niacin, cobalamin and magnesium)

^1^ Average duration of migraine attacks

Abbreviations: *MI* Migraine index, *HDR* Headache diary result, *MHIS* Migraine headache index score

**Fig. 2:**
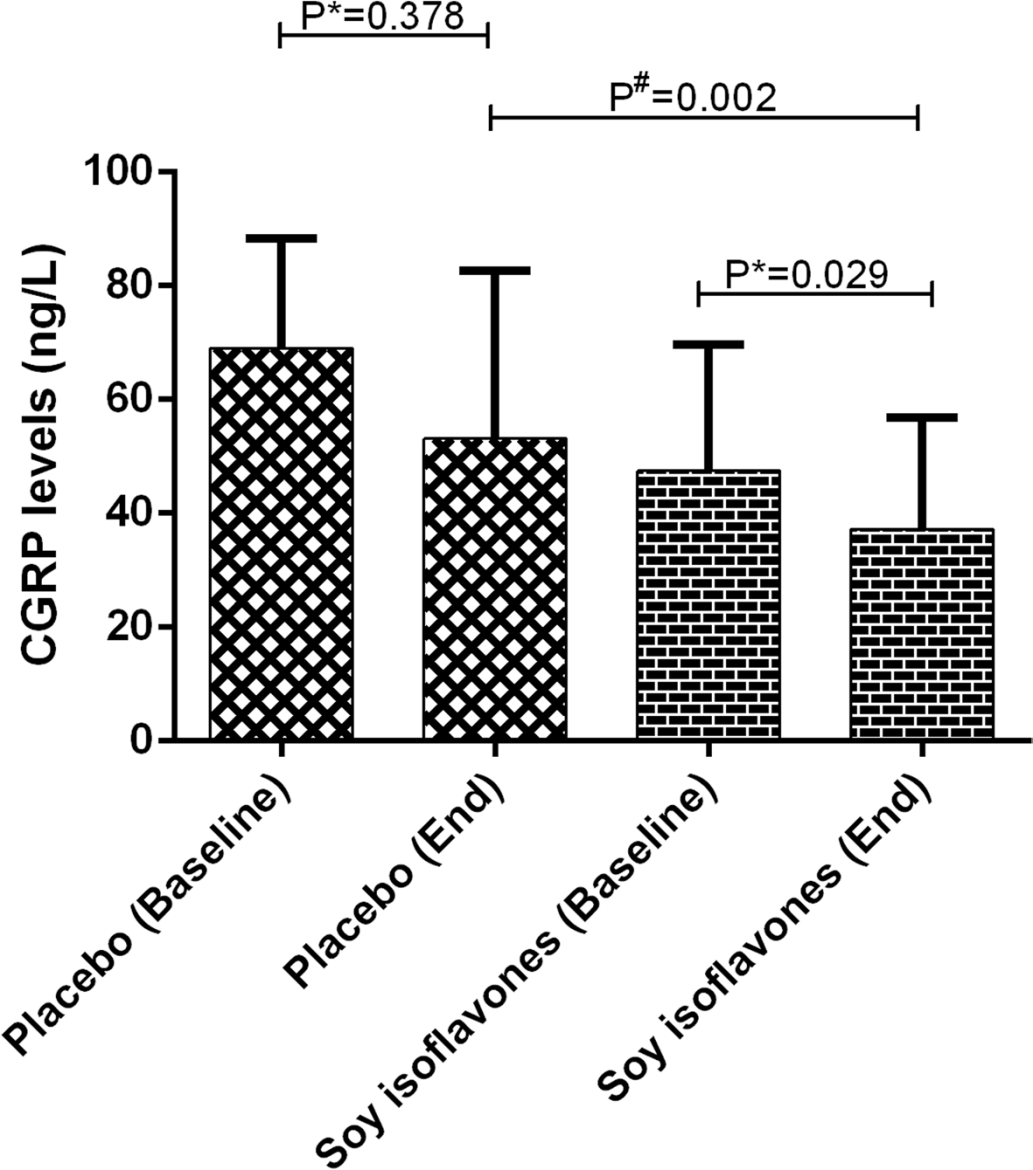
The effect of soy isoflavones supplementation on CGRP level. ^*^ *P*-value based on paired t-test was used to compare pre-post tests. ^#^ *p*-value Obtained from analysis of covariance in the adjusted models (adjusted for baseline value and certain dietary factors such as vitamin D, thiamin, riboflavin, niacin, cobalamin and magnesium)

Eight weeks of soy isoflavones supplementation resulted in a significant increase in migraine quality of life (MSQ score) (mean change: 16.76 ± 10.15 versus 2.52 ± 13), although, all of the demines related to mental status (stress, depression and anxiety) did not any significant improvement following soy isoflavones supplementation (*P* > 0.05) (Table 4). Soy isoflavones supplementation also resulted in a significant reduction in CGRP levels (mean change: -12.18 ± 35.73 versus -8.62 ± 64.24, *P* = 0.002) (Fig. 2) compared to placebo.

**Table 4:** The effects of 8 weeks’ soy isoflavones on quality of life and mental status of migraine patients

Variable		Soy isoflavones (*n *= 44)				Placebo (*n* = 44)				P ^b^	P ^c^
		Before	After	Change	P ^a^	Before	After	Change	P ^a^		
Quality of life (MSQ score)		54.49 ± 13.28	71.25 ± 10.15	16.76 ± 10.15	< 0.001	50.95 ± 21.46	53.48 ± 21.68	2.52 ± 13	0.205	< 0.001	< 0.001
Mental status	Depression	14.72 ± 9.17	14.75 ± 9.19	0.02 ± 1.63	0.927	17.31 ± 10.80	17.06 ± 10.42	-0.25 ± 2.78	0.555	0.272	0.978
	Anxiety	14.65 ± 6.97	14.56 ± 7.07	-0.09 ± 1.81	0.741	16.75 ± 9.55	16.50 ± 9.23	-0.25 ± 1.84	0.374	0.274	0.922
	Stress	18.75 ± 7.61	18.11 ± 8.35	-0.63 ± 3.43	0.225	24.29 ± 10.29	23.65 ± 10.12	-0.63 ± 3.67	0.258	0.006	0.957

Variables expressed as mean ± SD

a Paired t-test was used to compare pre-post tests

b Independent sample t-test was used to compare groups two by two

c Obtained from analysis of covariance in the adjusted models (adjusted for baseline value and certain dietary factors such as vitamin D, thiamin, riboflavin, niacin, cobalamin and magnesium)

## Discussion

The current study evaluated the effect of soy isoflavones supplementation on migraine characteristics and clinical indices of migraine attacks focusing for the first time on CGRP, quality of life, depression, stress and anxiety of the women suffering from migraine. The findings indicated that consumption of 50 mg/day soy isoflavones supplementation for 8 weeks led to significant reduction in frequency, duration, and clinical indices of migraine (MI, HDR and MHIS) and improved quality of life and CGRP levels; however, severity of migraine headache and mental status including depression, stress and anxiety were not affected by our supplementation.

To the best of our knowledge, there has been no RCT assessing the effect of soy isoflavones supplementation on migraine headache. However, soy isoflavones supplementation has been reported to be beneficial in reducing migraine attacks and severity. For example, soy isoflavones supplement including 56 mg/day genistein in combination with 20 mg/day daidzein in the first three days of menstruation days during 3- month reduced the frequency and severity of headache in women with menstrual migraine attacks [26]. However, this study used soy isoflavones supplement in a special type of migraine headache, making it difficult to compare the results with our study. Also, in other clinical trial that evaluated the effects of 60 mg soy isoflavones combined with specific herbs (100 mg *dong quai*, and 50 mg *black cohosh*) over 24 weeks period reduced menstrual migraine frequency and intensity [19]. This study used soy isoflavones in combination with other ingredients, making it difficult to differentiate the effect of soy isoflavones. Literature showed an inverse association between number of migraine attacks and estrogen levels in menstruation period and offer some protection effects of estrogen against migraine headache attacks [27].

This study could justify the effect of soy isoflavones on quality of life observed by decreasing the frequency and duration following supplementation. Previous studies had mentioned the decrease in number of migraine attacks and improvement in other migraine characteristic including severity and duration of headache upgrading in level of quality of life in these patients [28].

The findings of this study also suggested a reduction in the serum levels of CGRP following soy isoflavones supplementation. Previous studies had demonstrated that cytokines and CGRP [29] might be involved in the pathogenesis of migraine. On the other hand, there was a correlation between neurologic inflammation and CGRP release in migraine. Also, CGRP transcription could be stimulated by endogenous inflammatory molecules which increased the CGRP promoter activity and actuates MAPK pathway Release of CGRP from trigeminal nerve could stimulate inflammatory and immune responses. Pro-inflammatory cytokines might also activate nociceptors in the central and peripheral nervous system leading to the initiation of migraine pains [30].

CGRP is considered as a pain mediator, and it is expected that a reduction in CGRP serum levels leads to a clinical improvement in the migraine attacks in terms of frequency and duration during the supplementation. Moreover, literature search has showed a beneficial effects of soy isoflavones and soy products on inflammatory cytokines. Although, there has been no research assessing the effect of soy isoflavones on CGRP but other intervention with anti-inflammatory features have been helpful in CGRP levels [31].

Studies have shown that genistein as one of soy isoflavones has a tyrosine kinase inhibitor characteristic Tyrosine kinase can play a role in the pathophysiology of migraine with reduce in serotonin levels which is identified as a vasoconstrictor and its deficiency has been reported to play a role in pathophysiology of migraine [32]. To confirm that serotonin is involved in the pathogenesis of migraine, it can be noted that the triptans which are effective in the treatment of migraine are agonist at serotonin receptors.

### Strengths and limitations

The present study had some strength points needed to be taken into account. The study was the first to use an RCT design to investigate the effect of soy isoflavones on migraine characteristics, mental status and quality of life in a relatively large sample of women with migraine. Dietary intakes, physical activity and other confounding variables such as anthropometric indices, blood presser parameter were controlled during the study period. The study also did not use a run-in period as it might have affected the study’s external validity due to the possibility of excluding participants during the run-in period while an ITT approach was used in the data analysis of this study, a moderate to high dropout rate (5 in total) observed limited our power of analysis. The sample size of our study was small, although it was larger than previous studies. Also, clinical trials investigating inflammatory biomarkers and conduct in larger sample size in these patients were recommended to support the findings of this study.

Based on literature review dietary intakes have been linked to migraine headaches[13]. Although, we assessed dietary intakes via 3-day dietary surveys at the beginning and end of the trial. But we suggest a continuous and dynamic dietary surveys during the intervention period.

Although, there is a differences among participant habitual and isoflavones intake but we have not any assessment and compliance was assessed base on formula. Cytokine and serotonin levels is very important in clarifying pure effect of soy isoflavones on migraine characteristic although we did not check it. There is a differences among participant in cultural and dietary habits in the face of migraine control around the world which should be taken into account when interpreting the results of our study.

## Conclusions

Soy isoflavones could significantly reduce migraine attacks characteristics including headache, frequency and duration and clinical indices such as MI, HDR and MHIS in migraine patients. CGRP levels and quality of life also improved at the end of the study. However, mental status dimensions including depression, stress and anxiety were not affected. Further studies are needed to confirm these findings in the future.

## Data Availability

Not applicable.
